# Metal-organic framework-coated magnetite nanoparticles for synergistic magnetic hyperthermia and chemotherapy with pH-triggered drug release

**DOI:** 10.1080/14686996.2019.1682467

**Published:** 2019-10-24

**Authors:** Jiajie Chen, Jiaxing Liu, Yaping Hu, Zhengfang Tian, Yufang Zhu

**Affiliations:** a School of Materials Science and Engineering, University of Shanghai for Science and Technology, Shanghai, P. R. China; b Hubei Key Laboratory of Processing and Application of Catalytic Materials, College of Chemical Engineering, Huanggang Normal University, Huanggang, Hubei, P. R. China; c State Key Laboratory of High Performance Ceramics and Superfine Microstructure, Shanghai Institutes of Ceramics, Chinese Academy of Sciences, Shanghai, China

**Keywords:** Multifunctional nanoparticles, metal-organic frameworks, magnetic hyperthermia, chemotherapy, synergistic effect

## Abstract

In nanoplatform-based tumor treatment, combining chemotherapy with hyperthermia therapy is an interesting strategy to achieve enhanced therapeutic efficacy with low dose of delivery drugs. Compared to photothermal therapy, magnetic hyperthermia has few restrictions on penetrating tissue by an alternating magnetic field, and thereby could cure various solid tumors, even deep-tissue ones. In this work, we proposed to construct magnetic nanocomposites (Fe_3_O_4_@PDA@ZIF-90) by the external growth of metal-organic framework ZIF-90 on polydopamine (PDA)-coated Fe_3_O_4_ nanoparticles for synergistic magnetic hyperthermia and chemotherapy. In such multifunctional platform, Fe_3_O_4_ nanoparticle was utilized as a magnetic heating seed, PDA layer acted as an inducer for the growth of ZIF-90 shell and porous ZIF-90 shell served as drug nanocarrier to load doxorubicin (DOX). The well-defined Fe_3_O_4_@PDA@ZIF-90 core-shell nanoparticles were displayed with an average size of ca. 200 nm and possessed the abilities to load high capacity of DOX as well as trigger drug release in a pH-responsive way. Furthermore, the Fe_3_O_4_@PDA@ZIF-90 nanoparticles can raise the local temperature to meet hyperthermia condition under an alternating magnetic field owing to the magnetocaloric effect of Fe_3_O_4_ cores. In the *in vitro* experiments, the Fe_3_O_4_@PDA@ZIF-90 nanoparticles showed a negligible cytotoxicity to Hela cells. More significantly, after cellular internalization, the DOX-loaded Fe_3_O_4_@PDA@ZIF-90 nanoparticles exhibited distinctively synergistic effect to kill tumor cells with higher efficacy compared to chemotherapy or magnetic hyperthermia alone, presenting a great potential for efficient tumor therapy.

## GRAPHICAL ABSTRACT

**Figure:**
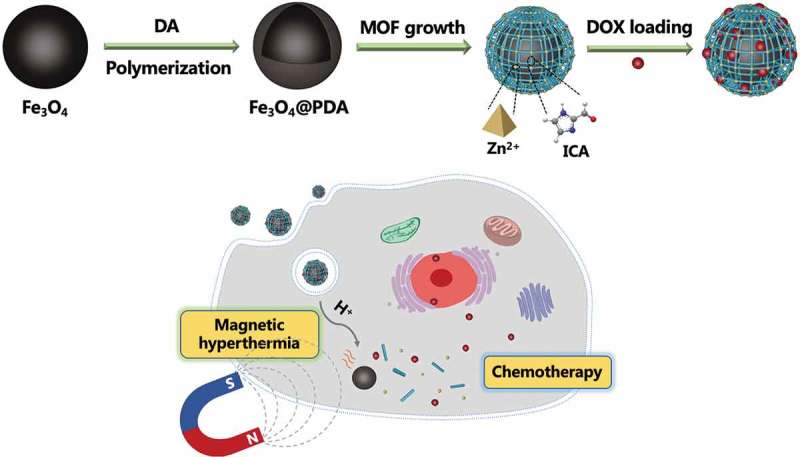

## 1. Introduction

Chemotherapy remains the most universally selected modality in clinical tumor therapy, but it suffers from the limitations including multidrug resistance and serious side effect, and thereby results in unsatisfied therapeutic efficacy. Recently, much efforts have been devoted to developing versatile nanoplatforms for integrating chemotherapy with other therapeutic forms, which could achieve synergistic effects for overcoming the drawbacks of chemotherapy and maximizing therapeutic efficacy [1–6]. Among these therapeutic modalities, thermal therapy, which causes local temperature above 40°C to damage tumor cells and tissues directly and sensitize tumors to chemotherapeutic drug, has been often introduced for synergistic therapy because of its satisfying antitumor efficacy and neglectable risk of recurrence [7]. Therefore, combining chemotherapy with thermal therapy into one single nanoplatform could realize satisfactory therapeutic efficacy with low dose of chemotherapeutic drugs, and thereby reduce the side effects.

For example, Chen et al. proposed to fabricate polypyrrole@metal-organic framework (PPy@MOF) nanocomposites, in which PPy core as photothermal agent and MIL-100 shell as carrier to load doxorubicin (DOX), for synergistic photothermal and chemotherapy [8]. Li et al. also fabricated a nanohybrid (Bi@mSiO_2_-PEG) by coating mesoporous silica on bismuth nanoparticles to load DOX for synergistic photothermal and chemotherapy [9]. Yang et al. reported the fabrication of a mesoporous CoFe_2_O_4_@PDA@ZIF-8 nanocomposite for DOX and camptothecin (CPT) multidrug chemo- and photothermal synergistic therapy [10]. More interestingly, Wang et al. utilized native high-density lipoproteins (HDLs) as whole components to integrate indocyanine green (ICG), tumor-penetrating peptide, and paclitaxel chemotherapeutic drug (pHDLs/PTX-ICG) for synergetic chemo-, photothermal, and photodynamic therapy [11]. Although nanoplatform-mediated photothermal therapy (PTT) possesses several advantages including high selectivity, remote controllability, and noninvasion, it is restricted by near infrared (NIR) light’s poor penetration depth and photothermal agents’ nonideal conversion efficiency.

In addition to photothermal therapy, magnetic hyperthermia (MHT) is another interesting nanoplatform-mediated thermal therapy, in which an alternating magnetic field (AMF) is employed to active magnetic materials for heat generation [12]. Compared to PTT, MHT has few restrictions on penetrating tissue by electromagnetic waves, and thereby could cure various solid tumors, even deep-tissue ones [13]. MHT has been considered as an efficient adjuvant to chemotherapy for tumor treatment and showed better synergistic effect with chemotherapy than other forms of hyperthermia [14]. More importantly, MHT has also been proved to induce individual’s own antitumor immune responses [15]. So far, superparamagnetic nanoparticles have been reported for MHT of tumors owing to their excellent magnetic performances and good biocompatibility, such as superparamagnetic iron oxide nanoparticles (SPIONs) [16–19] and superparamagnetic gold-nanoparticle clusters (SPAuNCs) [20]. Therefore, the construction of magnetic nanocomposites for synergistic magnetic hyperthermia with chemotherapy is beneficial for improving tumor therapeutic efficiency, which has obtained more and more attention [21]. In 2013, Kim et al. designed a composite nanofiber composed of a temperature-responsive copolymer (poly(NIPAAm-*co*-HMAAm)) with magnetic nanoparticles (a mixture of magnetite and maghemite) and DOX for reversibly magnetism-controlled drug release and synergistic chemotherapy and hyperthermia [22]. N’Guyen et al. proposed an effective strategy to decorate various drugs on SPIONs for controllable drug release and enhanced hyperthermia therapy [23]. Our group also reported several magnetic nanocomposites for potential magnetic hyperthermia and chemotherapy, such as Fe_3_O_4_-encapsulated mesoporous silica [24–26].

Recently, metal-organic frameworks (MOFs), as a particular type of hybrid porous materials assembled by metal ions or the secondary building units (SBUs) with organic ligands, have been exploited for various biomedical applications owing to their enormous surface area, high porosity, and adjustable structure and constitute, functional diversity as well as biocompatibility and biodegradability. Among them, as a class of biocompatible MOFs with acid-degradation, zeolitic imidazolate frameworks (ZIFs) are promising as pH-responsive drug carriers for chemotherapy, such as ZIF-8 and ZIF-90 nanoparticles [27–29]. In general, the pH values in the tumor microenvironments are often lower than those of normal tissues and bloodstream, such pH-responsive carriers can achieve specific tumor-associated drug release, resulting in efficient tumor therapy and high biosafety [30]. Particularly, ZIF-90 is coordinated by Zn^2+^ and imidazole-2-carboxaldehyde (ICA), and the aldehyde groups in the frameworks and high porosity of ZIF-90 could provide adequately interactions with various functional molecules, like chemotherapeutic drugs, enzymes et al. [31,32], and thereby improve the loading and reduce premature leakage. Fang et al. selected ZIF-90 as drug nanocarrier to delivery 5-fluorouracil (5-Fu) with slow drug leakage, and then investigated the AMF-triggered 5-Fu release as well as magnetic resonance (*T1* or *T2*) of the superparamagnetic nanoparticles embedded ZIF-90 [33]. More interestingly, Zhang et al. decorated DOX on the surface of ZIF-90 nanoparticles through covalent linking between amino groups and aldehyde groups, and encapsulated 5-Fu into the pores of the ZIF-90 frameworks for delivering two types of chemotherapeutic drugs to tumor cells with pH-responsive release [34].

Therefore, it can be speculated that combining magnetic nanoparticles with ZIF-90 to form core-shell nanocomposites could endow the nanocomposites with the functions of magnetic hyperthermia and chemotherapy with pH-triggered drug release, and thereby achieve synergistic therapeutic efficacy for tumor therapy. However, fabrication of well-defined nanoparticle@MOF core-shell composites with an ideal structure still faces challenges owing to its complexity and poor controllability. For example, Shieh’s group has succeeded in the construction of hybridized particles with hierarchical structure by growth of ZIF-8 microparticles/nanoparticles on the surface of amine-functionalized siliceous mesocellular foams (MCF), which could be extended to coat different types of ZIFs on the external surface of other materials to form composites [35]. More interestingly, polydopamine (PDA), produced *via* the self-polymerization of dopamine molecules, has been reported to play significant roles in constructing nanoparticle@MOF core-shell composites [36,37]. The catechol groups in PDA with metal-chelating character are beneficial for heterogeneous nucleation and further growth of MOF on the outside of PDA-coated nanoparticles. On the other hand, the PDA layer improves the dispersion and colloidal stability, preventing nanoparticles from aggregation during the growth of MOF [36]. Therefore, superficial coating of PDA on magnetic nanoparticles before the growth of ZIF-90 shell is an intelligent choice to fabricate a magnetic nanoparticles/ZIF-90 core-shell nanocomposite.

**Scheme 1.**
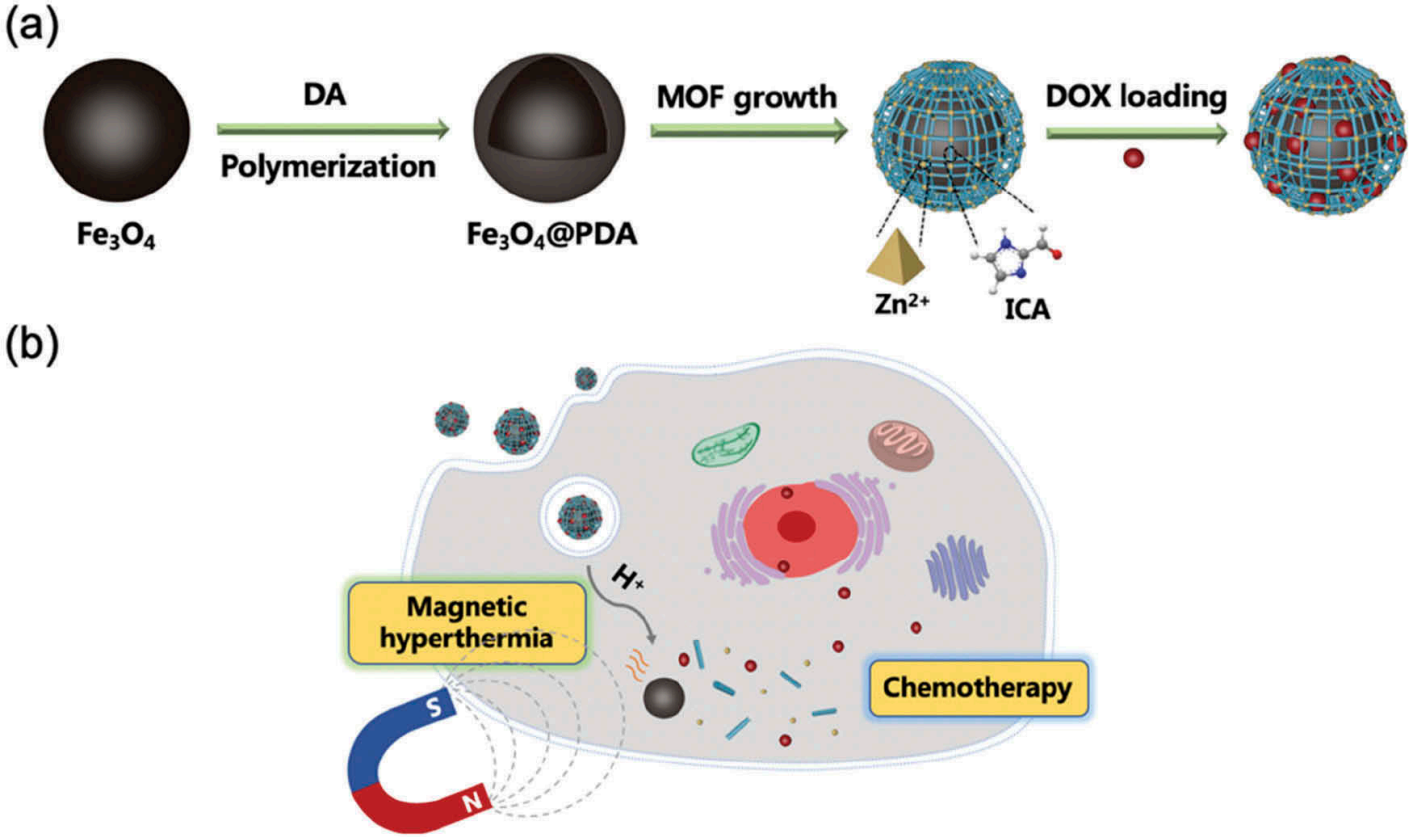
Schematic illustrations of (a) the synthesis of Fe_3_O_4_@PDA@ZIF-90 core-shell nanoparticles and drug loading as well as (b) the use of drug-loaded nanoparticles for synergistic magnetic hyperthermia and chemotherapy.

In this work, we proposed to construct magnetic Fe_3_O_4_/ZIF-90 core-shell nanocomposites as nanocarriers for potential tumor therapy with synergistic magnetic hyperthermia and chemotherapy. Herein, magnetic Fe_3_O_4_ nanoparticles were prepared through hydrothermal process, and then were capped by PDA layer along with dopamine self-polymerization. Subsequently, Zn^2+^ ions were chelated on the surface of PDA layer, and then induced ZIF-90’s superficial growth on PDA-coated Fe_3_O_4_ nanoparticles for forming Fe_3_O_4_@PDA@ZIF-90 core-shell nanoparticles. Doxorubicin (DOX) was selected to study the drug carrying and pH-triggered release behavior (Scheme 1a). After endocytosis by tumor cells, the drug-loaded Fe_3_O_4_@PDA@ZIF-90 nanoparticles could not only achieve chemotherapy due to drug release triggered by the acidic environment in tumor cells, but also induce magnetic hyperthermia under an AMF due to the magnetocaloric effect of Fe_3_O_4_ nanoparticles (Scheme 1b).

## 2. Experimental section

### 2.1. Materials

Anhydrous sodium citrate (C_6_H_5_Na_3_O_7_), zinc nitrate hexahydrate (Zn(NO_3_)_2_ · 6H_2_O), iron chloride hexahydrate (FeCl_3_ · 6H_2_O), urea (CH_4_N_2_O), poly(acrylamide) (PAM), tris(hydroxymethyl)methyl aminomethane (THAM), *N,N*-dimethylformamide (DMF), ethanol, and hydrochloric acid (HCl, ≥36%) were bought from Sinopharm Group Chemical Reagent Co., Ltd., China. Phosphate-buffered saline (PBS) tablets and doxorubicin hydrochloride (DOX) were bought from Sangon Bioengineering Co., Ltd., Shanghai, China. Dopamine hydrochloride (DA-HCl) and imidazole-2-carboxaldehyde (ICA) were obtained from Sigma-Aldrich Co., Ltd., Shanghai, China. Trioctylamine (TOA) was obtained from Aladdin Chemistry Co., Ltd., Shanghai, China. None of the chemicals were further purified before usage.

### 2.2. Characterization

FEI Quanta 450 field emission scanning electron microscope (SEM) with a test voltage of 30 kV and Tecnai G2 F30 transmission electron microscope (TEM) with an acceleration voltage of 300 kV were adopted to analyze the morphologies and structures of nanoparticles. Dynamic light scattering (DLS) analysis was performed to measure the hydrodynamic sizes of nanoparticles by a Zetasizer Nano ZS90 (Malvern Instruments, UK). D8 ADVANCE powder diffractometer (Bruker, Germany) was employed to obtained the powder X-ray diffraction (XRD) patterns under Cu Kα radiation (λ = 0.154 nm). SPECTRUM 100 Fourier transform infrared spectrometer (PerkinElmer, USA) was used to record the FTIR spectra in KBr plates. Thermogravimetric analysis (TGA) was carried out by PerkinElmer Pyris 1 thermal analyzer heated from 50 to 800 °C at a rate of 10°C/min and a N_2_ flow rate of 20 mL/min. NanoDrop 2000C ultraviolet-spectrophotometer (Thermo Fisher Scientific, USA) was selected to obtain the UV-Vis absorption spectra.

### 2.3. Synthesis of magnetic Fe_3_O_4_ nanoparticles

First of all, magnetite nanoparticles were prepared by a simple one-pot hydrothermal way [38]. The 70 mL aqueous solution containing 1.5435 g C_6_H_5_Na_3_O_7_, 0.63 g CH_4_N_2_O, 0.71 g FeCl_3_ · 6H_2_O, and 0.525 g PAM was sealed in a Teflon-lined steel-autoclave and kept at 180°C for 10 h. After the finish of reaction, the black precipitate was collected by a magnet and rinsed with deionized water for three times. Finally, the synthesized Fe_3_O_4_ nanoparticles were vacuum-dried at 60°C overnight.

### 2.4. Synthesis of Fe_3_O_4_@PDA@ZIF-90 nanoparticles

For coating polydopamine (PDA) layer, the synthesized Fe_3_O_4_ nanoparticles (68 mg) were dispersed in 200 mL Tris-HCl buffer (10 mM, pH 8.5), and DA (136 mg) were added into solution later under stirring. After reaction for 2 h, the precipitate was collected by a magnet and rinsed with deionized water for three times. The PDA-coated Fe_3_O_4_ (Fe_3_O_4_@PDA) nanoparticles were dispersed in 6 mL deionized water for further usage.

For growth of ZIF-90 on Fe_3_O_4_@PDA nanoparticles, Fe_3_O_4_@PDA nanoparticle solution (1 mL) was injected into 20 mL ethanol under vigorous stirring. On the other hand, the 60 mL ethanol containing Zn(NO_3_)_2_ · 6H_2_O (800 mg) were prepared, and subsequently added into the Fe_3_O_4_@PDA ethanol solution with vigorous stirring for 12 h. After that, the Zn^2+^-chelated nanoparticles (Fe_3_O_4_@PDA-Zn) were magnetically collected and rinsed with deionized water, and dispersed in 50 mL DMF for further usage.

Next, ICA solution (96 mg/50 mL DMF), Zn(NO_3_)_2_ solution (99 mg/50 mL DMF), and TOA solution (0.4 mL/25 mL DMF) were prepared, respectively. Then, 10 mL ICA solution was poured into the obtained Fe_3_O_4_@PDA-Zn solution under stirring. After 1 h, 10 mL Zn(NO_3_)_2_ solution, 5 mL TOA solution and 10 mL ICA solution were poured in turn at regular intervals until all the residual solutions were added and continuously reacted for 12 h. Finally, Fe_3_O_4_@PDA@ZIF-90 nanoparticles were magnetically collected and rinsed with deionized water for three times before drying in vacuum at 60°C overnight.

### 2.5. Drug loading and release

30 mg of Fe_3_O_4_@PDA@ZIF-90 nanoparticles were dispersed in 12 mL of DOX aqueous solution (0.5 mg/mL) and then the mixture was stirred for 24 h. After collecting by centrifugation, the drug-loaded nanoparticles were rinsed with deionized water to remove the unloaded DOX molecules. The UV-Vis absorbance at 482 nm of the supernatant was recorded to determine the concentration of DOX. Then the loading content and loading efficiency of DOX were calculated. The equation was used to calculate the drug-loading efficiency as follows: DOX Loading (%) = (weight of loaded DOX/original weight of DOX) × 100%.

For analyzing drug release profiles, the DOX-loaded Fe_3_O_4_@PDA@ZIF-90 nanoparticles (2 mg) were soaked in 0.5 mL of PBS solution with different pH (4.5, 6.0, and 7.4). The systems were kept at 37°C with a gentle shaking. At certain intervals, 2 μL of medium was collected to determine the content of DOX for three times, and the remaining dialysate supplemented with 6 μL fresh PBS solution. According to the standard curve of DOX solution, the concentration of the released DOX was detected by UV-Vis analysis at 482 nm. The equation was used to calculate the release efficiency of DOX as follows: DOX Release (%) = (weight of released DOX/weight of loaded DOX in nanoparticles) × 100%.

### 2.6. Magnetic heating test

Magnetic heating ability was characterized by DM100 series (nanoScale Biomagnetics, Spain). An AMF was set at 409 kHz of frequency and 180 Gauss of field. To evaluate magnetic heating ability of different nanoparticles, Fe_3_O_4_, Fe_3_O_4_@PDA, Fe_3_O_4_@PDA@ZIF-90 nanoparticles in water with the total particle concentration of 5 mg/mL were prepared. Subsequently, 1 mL liquid was added into test bottle before treatment with an AMF for 20 min. The temperature changes of suspension were recorded in real time by a fiber optic thermometer.

### 2.7. Cellular uptake

Here, Hela cell line, which was provided by Stem Cell Bank, Chinese Academy of Sciences, was used for *in vitro* experiments. Hela cells were cultured in a humidified incubator (Thermo Fisher Scientific, USA) at 37 °C with a 5% CO_2_ atmosphere in Minimum Eagle’s Medium (MEM, GIBCO, Invitrogen) added with fetal bovine serum (FBS) (10%) and penicillin-streptomycin solution (1%). To observe cellular uptake of the Fe_3_O_4_@PDA@ZIF-90/DOX nanoparticles, 1 × 10^5^ Hela cells were seeded in a 35-mm culture dish before maintaining in the incubator for 24 h. Hela cells were sequentially incubated with fresh culture medium containing 100 μg/mL Fe_3_O_4_@PDA@ZIF-90/DOX nanoparticles for 4 h and subsequently washed with PBS solution twice. After that, the cell nuclei were stained with a solution of 4ʹ,6-diamidino-2-phenylindole (DAPI) in methanol for 15 min at 37°C before using methanol to wash several times. In the end, the Hela cells were fixed, and observed on confocal laser scanning microscopy (CLSM, Leica, SP5, Germany).

### 2.8. Cytotoxicity assay

To evaluate the cytotoxicity of Fe_3_O_4_@PDA@ZIF-90 nanoparticles, the standard 3-(4,5-dimethylthialzol-2-yl)-2,5-diphenyltetra-zolium bromide (MTT) assay was implemented for Hela cells. Hela cells were firstly seeded in 96-well microplate at 1 × 10^4^ cells per well and then incubated at 37°C for 24 h. After that, 0, 25, 50, 100, and 200 μg/mL Fe_3_O_4_@PDA@ZIF-90 nanoparticles in culture medium were added and incubated with cells for anther 24 h at 37°C. For standard MTT assay, 10 μL of MTT (5mg/mL in PBS) solution was added to each well before the incubation for another 4 h. After the supernatants were removed carefully, 100 μL of DMSO was added and the 96-well microplate was shaken for 10 min before the absorbance at 490 nm was recorded by a microplate reader (Bio-Rad 680, California, USA).

### 2.9. In vitro synergistic therapy evaluation

For synergistic magnetic hyperthermia and chemotherapy, Hela cells in 96-well microplate were treated with culture medium containing 13.8 μg/mL free DOX (the same amount of drug as in the 100 μg/mL Fe_3_O_4_@PDA@ZIF-90/DOX) as well as 100 μg/mL Fe_3_O_4_@PDA, Fe_3_O_4_@PDA@ZIF-90 and Fe_3_O_4_@PDA@ZIF-90/DOX at 37°C for 4 h. For magnetic hyperthermia, different groups including the control group were treated with AMF for 10 min. To enhance the therapeutic effect, these different groups were treated with AMF twice, i.e. after treatment under magnetic field for 10 min, the cells moved to the incubator for another 8 h culture, and then treated with AMF for another 10 min. Finally, the cells were incubated for anther 2 h to evaluate cell viability. Cell viability tests were carried out by MTT assay as mentioned above.

## 3. Results and discussion

### 3.1. Synthesis and characterization

**Figure 1.**
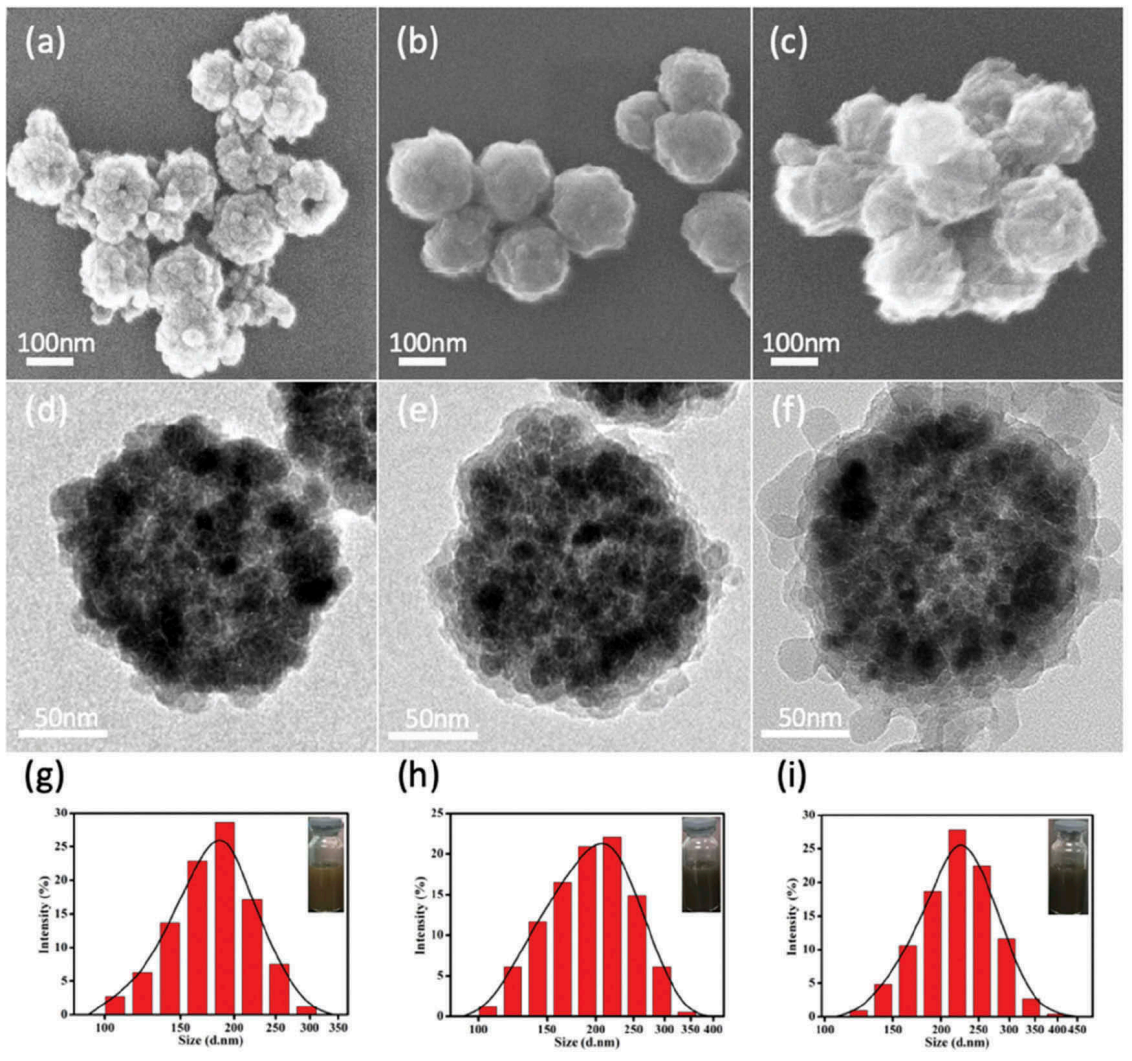
SEM, TEM graphs and DLS size distributions of (a,d,g) the synthesized Fe_3_O_4_, (b,e,h) Fe_3_O_4_@PDA, and (c,f,i) Fe_3_O_4_@PDA@ZIF-90 nanoparticles (inset: photographs of the nanoparticles dispersed in water) .

Fe_3_O_4_@PDA@ZIF-90 nanoparticles were synthesized by coating Fe_3_O_4_ nanoparticles with PDA layer and following superficial growth of ZIF-90 on Fe_3_O_4_@PDA (Scheme 1a), which was described minutely in the experimental section. The morphologies and structures of Fe_3_O_4_, Fe_3_O_4_@PDA and Fe_3_O_4_@PDA@ZIF-90 nanoparticles are shown in Figure 1. Fe_3_O_4_ nanoparticles were formed by the aggregation of Fe_3_O_4_ small crystals, presenting a well-defined spherical structure with a diameter of approximately 170 nm (Figure 1(a,d)). After self-polymerization of dopamine molecules, thin polydopamine (PDA) layers were uniformly covered over the surface of Fe_3_O_4_ nanoparticles (Figure 1(e)). Interestingly, the PDA coating provided nanoparticles with smooth surface and good dispersibility, which might avoid the aggregation of the magnetic particles in the ZIF-90 growth process. On the other hand, the catechol groups on PDA coating could chelated with Zn^2+^ for heterogeneous nucleation and ZIF-90’s superficial growth on PDA-coated nanoparticle [10]. As shown in the Figure 1(f), 20–30 nm of ZIF-90 nanoparticles were deposited on PDA-coated Fe_3_O_4_ nanoparticle to form core-shell nanoparticles, and the average particle size of Fe_3_O_4_@PDA@ZIF-90 nanoparticles was about 200 nm, which might results in effective cellular internalization by the enhanced permeability and retention (EPR) effect [39]. Further, the similar information about the hydrodynamic diameters of these nanoparticles was revealed by DLS analysis, and it also showed all the nanoparticles possessed narrow size distribution and well dispersion (Figure 1(g–i).

**Figure 2.**
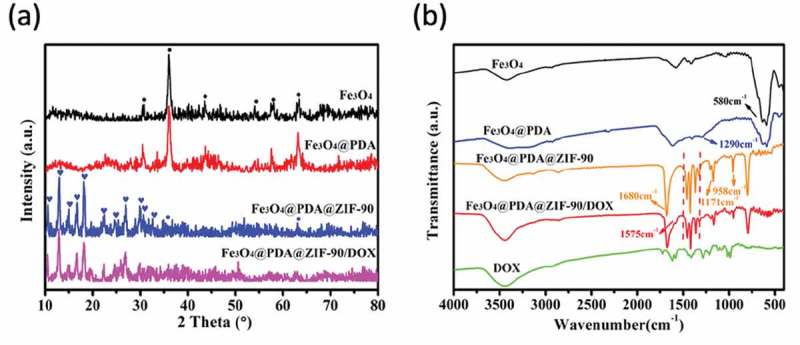
(a) Powder XRD patterns and (b) FTIR spectra of different nanoparticles.

On the other hand, the powder XRD patterns and FTIR spectra were also used to further confirm the construction of Fe_3_O_4_@PDA@ZIF-90 nanoparticles. As illustrated in the XRD patterns (Figure 2(a)), the peaks of Fe_3_O_4_ observed at 2*θ* = 30.7°, 36.0°, 43.5°, 54.0°, 58.0°, and 63.4° were assigned to the (220), (311), (400), (422), (511), and (440) planes (JCPDS Card No. 19–0629). The characteristic peaks of Fe_3_O_4_@PDA nanoparticles were same as Fe_3_O_4_ nanoparticles due to the amorphous PDA structure, suggesting that the coating process of PDA layer did not influence the original crystallinity of Fe_3_O_4_ nanoparticles. After the growth of ZIF-90 on Fe_3_O_4_@PDA nanoparticles, the additional characteristic peaks at 2*θ* = 10.5°, 12.9°, 14.9°, 16.6°, 18.0°, 22.3°, 24.5°, 26.8°, 29.8°, 30.6°, and 32.5° were correspond to the (200), (112), (022), (013), (222), (114), (223), (134), (044), (244), and (235) planes of crystalline ZIF-90. Both of the emergence of the characteristic peaks of ZIF-90 and the weakness of the characteristic peaks of Fe_3_O_4_ indicated the successful growth of ZIF-90 on Fe_3_O_4_@PDA nanoparticle. In FTIR spectra (Figure 2(b)), stretching vibration at 580 cm^−1^ was assigned to the Fe-O bonds in Fe_3_O_4_ nanoparticles. After polymerization of dopamine on Fe_3_O_4_ nanoparticle, one more peak at 1290 cm^−1^ was related to the C-O and C-N stretching vibration on PDA layer. The vibrations at 1680 cm^−1^ (C = O), 1350 ~ 1500 cm^−1^ (imidazole ring), 1171 and 958 cm^−1^ (C-N) were the special bonds of ZIF-90, and thereby confirmed the growth of ZIF-90 on the nanoparticles.

**Figure 3.**
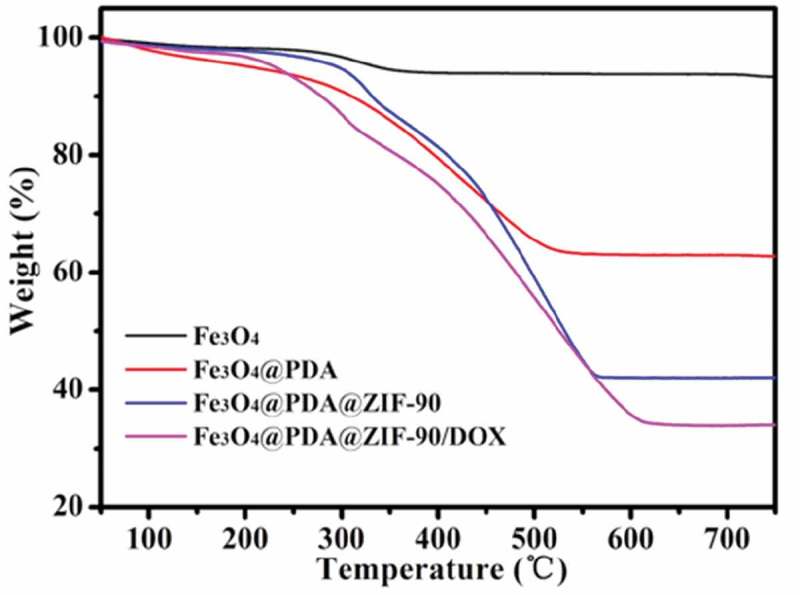
TGA of Fe_3_O_4_, Fe_3_O_4_@PDA, Fe_3_O_4_@PDA@ZIF-90, and Fe_3_O_4_@PDA@ZIF-90/DOX nanoparticles.

TGA was carried out to further characterize different nanoparticles (Figure 3). Before 200°C, the weight loss was the result of the ejection of the moisture. Compared with Fe_3_O_4_ nanoparticles, Fe_3_O_4_@PDA nanoparticles had significant weight loss starting at around 300°C owing to the decomposition of PDA. More weight loss of Fe_3_O_4_@PDA@ZIF-90 nanoparticles was observed in the range from 300 to 570°C, indicating the existence of ZIF-90 and its desirable thermal stability. On the basis of the weightlessness ratio, the mass ratios of Fe_3_O_4_, PDA, and ZIF-90 ingredients in the nanocomposites were calculated to be 44.4%, 19.7%, and 35.9%, respectively.

### 3.2. Drug loading and pH-triggered release profiles

**Figure 4.**
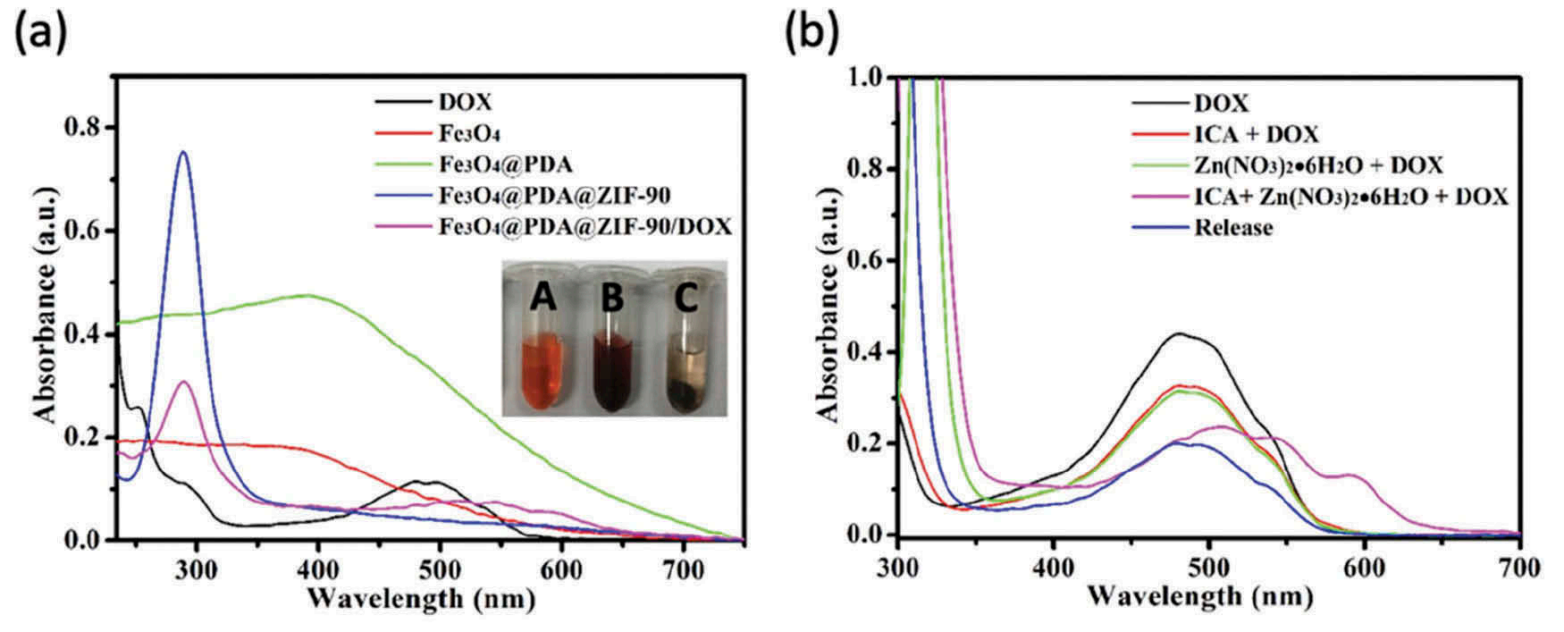
(a) UV-Vis absorption spectra of DOX and different nanoparticle aqueous solutions (inset: photographs of (A) DOX solution, (B) the mixture solution of DOX and Fe_3_O_4_@PDA@ZIF-90 nanoparticles, and (C) the mixture solution of DOX and Fe_3_O_4_@PDA@ZIF-90 nanoparticles after reaction for the whole day); (b) UV-Vis absorption spectra of different mixture solutions and the DOX solution after drug release from Fe_3_O_4_@PDA@ZIF-90 nanoparticles at pH 6.0.

Here, doxorubicin (DOX) was selected as an antitumor drug model to investigate its loading by Fe_3_O_4_@PDA@ZIF-90 nanoparticles and pH-triggered release behavior. The DOX loading of Fe_3_O_4_@PDA@ZIF-90 nanoparticles was analyzed by the UV-Vis absorption spectra (Figure 4(a)). A characteristic absorption band of DOX was observed at 482 nm. No characteristic absorption peaks in visible light region were observed from Fe_3_O_4_, Fe_3_O_4_@PDA, and Fe_3_O_4_@PDA@ZIF-90 nanoparticles, while a strong absorption band shift to λ = 508 nm for the DOX-loaded Fe_3_O_4_@PDA@ZIF-90 nanoparticles, indicating the loading of DOX in the Fe_3_O_4_@PDA@ZIF-90 nanoparticles. The color change from red to colorless for the mixture solution of DOX and Fe_3_O_4_@PDA@ZIF-90 nanoparticles after reaction for 24 h is shown in the inset from Figure 4(a), and the absorption of the supernatant after reaction was almost zero, which also illustrated the successful loading of DOX into Fe_3_O_4_@PDA@ZIF-90 nanoparticles. To further investigate the red-shift of characteristic absorption band of DOX after loading into Fe_3_O_4_@PDA@ZIF-90 nanoparticles, DOX was mixed with the precursors of ZIF-90 and the relevant UV-Vis absorption spectra were recorded. Interestingly, the characteristic absorption bands of the samples that DOX was mixed with ICA ligand or Zn(NO_3_)_2_ · 6H_2_O were same as the absorption band of DOX, but only the absorption band of the sample with the mixture of DOX, ICA ligand, and Zn(NO_3_)_2_ · 6H_2_O was red-shift to λ = 508 nm in Figure 4(b). Moreover, the characteristic absorption of the released DOX from Fe_3_O_4_@PDA@ZIF-90 nanoparticles at pH 6.0 was still at λ = 482 nm. These results indicated that the red-shift of absorption band might be the result of the interactions between DOX molecules and the functional groups in ZIF-90 frameworks, including π–π stacking interaction, hydrogen bonding, and covalent bonding. These intricate interactions between DOX and ZIF-90 shell were potentially beneficial for the prevention from premature drug release before arriving at target site. The covalent bonding of DOX in ZIF-90 shell was also proved in the FTIR spectra of the Fe_3_O_4_@PDA@ZIF-90/DOX nanoparticles (Figure 2(b)), where the characteristic bond emerged at 1575 cm^−1^ was correspond to the C = N bonds in result of the reaction between aldehyde groups of ZIF-90 and amino groups of DOX molecule. The DOX loading content and loading efficiency of Fe_3_O_4_@PDA@ZIF-90 nanoparticles were calculated to be as high as 160 μg/mg and 80%, which were attributed to the high porosity of MOF shell and intricate interactions between DOX and Fe_3_O_4_@PDA@ZIF-90 nanoparticles. After DOX loading, the characteristic peaks of the DOX-loaded nanoparticles in the XRD pattern were the same as the Fe_3_O_4_@PDA@ZIF-90 nanoparticles (Figure 2(a)), indicating that the loaded DOX was amorphous, and the DOX loading did not change the crystallinity of ZIF-90. Compare with the Fe_3_O_4_@PDA@ZIF-90 nanoparticles, the additional weight loss of the Fe_3_O_4_@PDA@ZIF-90/DOX nanoparticles from TGA was about 15% (Figure 3), which was consistent with the DOX loading content from UV-Vis analysis.

**Figure 5.**
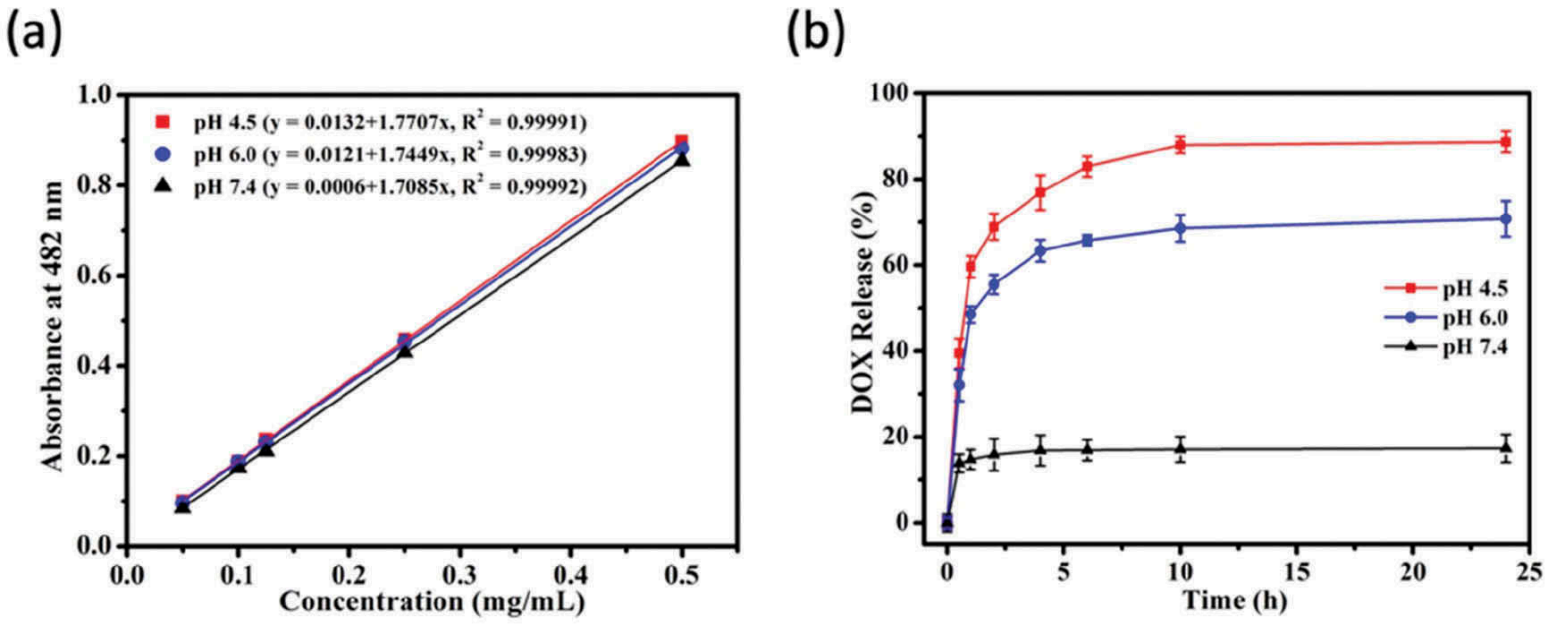
(a) The standard curve of DOX solution in different pH conditions; (b) the DOX release profiles of Fe_3_O_4_@PDA@ZIF-90 nanoparticles at pH 4.5, 6.0, and 7.4.

To study the pH-triggered DOX release from Fe_3_O_4_@PDA@ZIF-90 nanoparticles, the drug release profiles were tested at three disparate pH conditions (PBS, pH 7.4, 6.0 and 4.5), which were selected respectively to mimic the neutral environment in healthy cells, intratumoral mildly acidic environment, and even more acidic environments in the intracellular organelles such as endosomes and lysosomes [40]. The standard curves of DOX solution in these pH conditions were fitted in Figure 5(a) for reference. Consequently, as seen in the Figure 5(b), the DOX-loaded Fe_3_O_4_@PDA@ZIF-90 nanoparticles exhibited a slow drug release at pH 7.4 and only 17.3% of drug was discharged after 24 h. However, the DOX release was accelerated and the released contents were estimated to be 70.8% at pH 6.0 and 88.7% at pH 4.5 after 24 h, respectively. Comparatively, a slight decrease in pH from neutral condition significantly improved the drug release from Fe_3_O_4_@PDA@ZIF-90 nanoparticles. The pH-triggered drug release character of Fe_3_O_4_@PDA@ZIF-90 nanoparticles is attributed to the acid-degradation of ZIF-90 shell, i.e. ZIF-90 is stable under neutral condition but degradable under acidic condition, which caused by the dissociation of coordination bonds in ZIF-90. In this case, such pH-responsiveness of Fe_3_O_4_@PDA@ZIF-90 nanoparticles is beneficial for effective antitumor drug delivery and acidic tumor microenvironment-mediated chemotherapy with low side effects to normal cells.

### 3.3. Magnetic hyperthermia capacity

**Figure 6.**
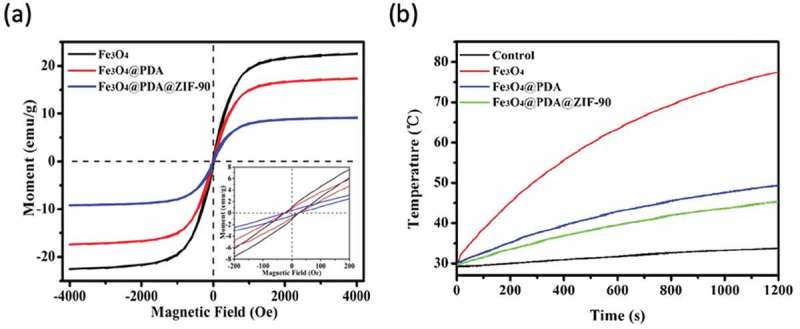
(a) Magnetization curves of different nanoparticles; (b) temperature variation curves of 5 mg/mL different nanoparticles in water under an AMF with 409 kHz and 180 Gauss for 20 min.

The magnetization curves of the Fe_3_O_4_, Fe_3_O_4_@PDA, and Fe_3_O_4_@PDA@ZIF-90 nanoparticles with the same total amount were tested by a vibrating sample magnetometer (VSM) at room temperature. Extremely small hysteresis loops were found from the Fe_3_O_4_@PDA and Fe_3_O_4_@PDA@ZIF-90 nanoparticles’ magnetization curves, which were similar to bare Fe_3_O_4_ nanoparticles (Figure 6(a)). The coercivity and the remanence of these nanoparticles were less than 25 Oe and 1 emu/g separately, indicating that the superparamagnetic character of Fe_3_O_4_ nanoparticles was still maintained during the PDA coating and ZIF-90 growth process. Furthermore, the saturation magnetization of Fe_3_O_4_, Fe_3_O_4_@PDA, and Fe_3_O_4_@PDA@ZIF-90 nanoparticles was 22.5, 17.3, and 9.2 emu/g, respectively. This variation of saturation magnetization was due to the decrease of Fe_3_O_4_ content in each sample. The relatively high magnetization revealed that such magnetic nanoparticles are of great potential for magnetic hyperthermia therapy.

Under an AMF, the temperature increases of superparamagnetic Fe_3_O_4_, Fe_3_O_4_@PDA, and Fe_3_O_4_@PDA@ZIF-90 nanoparticles with the same particle concentration of 5 mg/mL were recorded in Figure 6(b). After being treated with an AMF at 409 kHz and 180 Gauss for 20 min, magnetic Fe_3_O_4_ nanoparticles exhibited high boost of temperature and the temperature increased from 30 to 77.5°C, while slight temperature change was observed for water, indicating the excellent magnetic heating performance of magnetic Fe_3_O_4_ nanoparticles. Due to the decrease of Fe_3_O_4_ amount in the same concentration of Fe_3_O_4_@PDA and Fe_3_O_4_@PDA@ZIF-90 nanoparticles, a little lower temperature increases happened to these nanoparticles compared to Fe_3_O_4_ nanoparticles. The temperature increases reached to 49.4°C and 45.6°C for Fe_3_O_4_@PDA and Fe_3_O_4_@PDA@ZIF-90 nanoparticles, respectively. Obviously, the magnetic heating ability of the Fe_3_O_4_@PDA@ZIF-90 nanoparticles meets the requirement of hyperthermia temperature. Due to tumor cells/tissues possess a lower tolerance of temperature above 40°C than normal tissues, the Fe_3_O_4_@PDA@ZIF-90 nanoparticles could be utilized to ablate tumor cells and tissues under an AMF and sensitize them to chemotherapeutic drug. Under acidic tumor microenvironment, the dissociation of non-magnetic ZIF-90 shell might enhance the magnetic heating ability. On the other hand, magnetic heating could accelerate drug release due to the promotion of drug diffusion rate.

### 3.4. Cellular uptake

**Figure 7.**
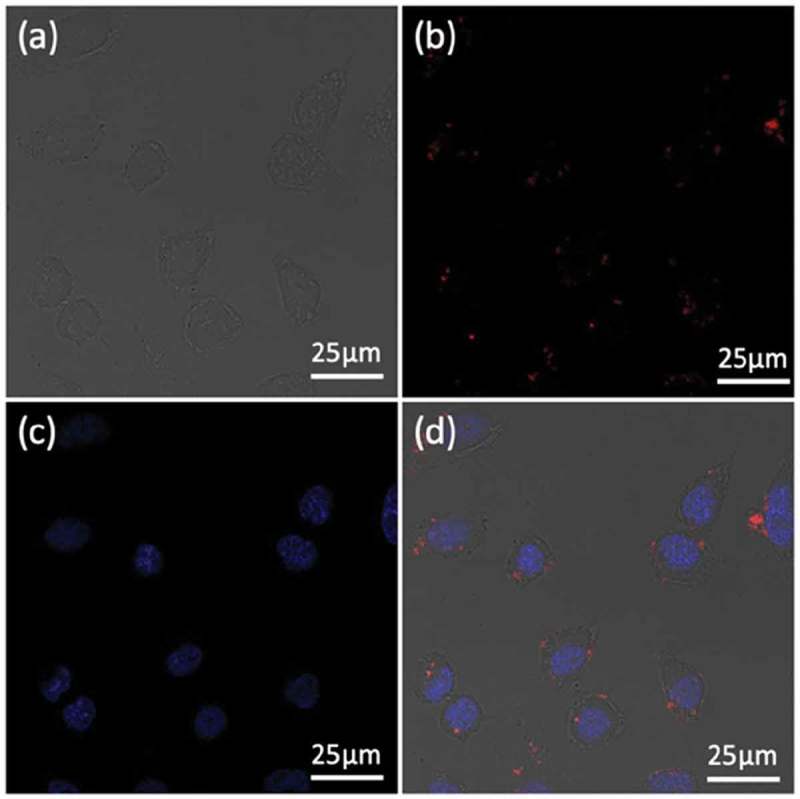
CLSM images of Hela cells after treatment with Fe_3_O_4_@PDA@ZIF-90/DOX nanoparticles for 4 h: (a) bright field; (b) the red fluorescence from DOX; (c) the blue fluorescence from DAPI; and (d) merged image.

To study the cellular localization of the Fe_3_O_4_@PDA@ZIF-90/DOX nanoparticles, Hela cells were incubated with the nanoparticles for 4 h and then observed by confocal laser scanning microscope (CLSM). Here, DAPI with blue fluorescence under excitation at 405 nm was adopted to stain the Hela cell nuclei, and the red fluorescence of the loaded DOX under excitation at 535 nm was selected to visualize the position of nanoparticles and the final fate of DOX. As seen in Figure 7, the red fluorescent signals primarily appeared in the Hela cells’ cytoplasm, confirming the successful internalization of the DOX-loaded Fe_3_O_4_@PDA@ZIF-90 nanoparticles into cells after incubation for 4 h. This result indicated that Fe_3_O_4_@PDA@ZIF-90 nanoparticles could not only enhance drug delivery into tumor cells and its release into organelles for chemotherapy, but also facilitate intracellular heat generation for magnetic hyperthermia, showing great potential for effectively synergistic antitumor therapy.

### 3.5. Cytotoxicity assay and in vitro synergistic therapy evaluation

**Figure 8.**
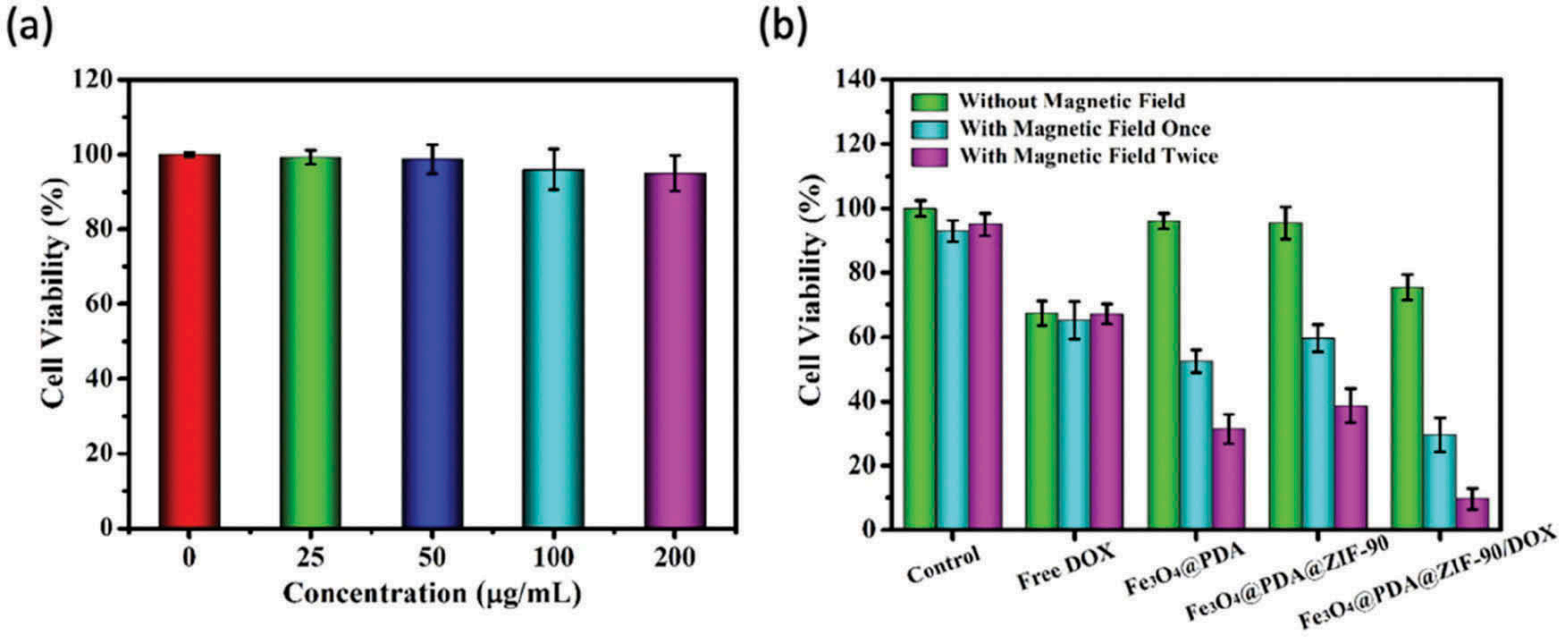
(a) Cell viability tested by MTT assay for Hela cells treated with Fe_3_O_4_@PDA@ZIF-90 nanoparticles with various concentrations for 24 h; (b) evaluation of therapeutic effect for Hela cells incubated with different nanoparticles and free DOX with or without treatment under an AMF.

The cytotoxicity of Fe_3_O_4_@PDA@ZIF-90 nanoparticles was evaluated before synergistic therapy. For it, using the Fe_3_O_4_@PDA@ZIF-90 nanoparticles at various concentrations to treat Hela cells for 24 h and adopting MTT assay to analyze the cell viability (Figure 8(a)). All treated cells showed negligible fatality, even the concentration of nanoparticles up to 200 μg/mL, suggesting that the Fe_3_O_4_@PDA@ZIF-90 nanoparticles have good biocompatibility and negligible cytotoxicity. To further evaluate the synergistic magnetic hyperthermia and chemotherapy of the DOX-loaded Fe_3_O_4_@PDA@ZIF-90 nanoparticles, the cytotoxicity of 13.8 μg/mL of free DOX and 100 μg/mL of different nanoparticles with and without the treatment under an AMF were also analyzed by MTT assay. Figure 8(b) revealed that the cell viabilities of Hela cells treated with DOX-free Fe_3_O_4_@PDA and Fe_3_O_4_@PDA@ZIF-90 nanoparticles were basically the same as that of the control group. Both of free DOX and DOX-loaded Fe_3_O_4_@PDA@ZIF-90 nanoparticles exhibited certain chemotherapeutic effect. Compared to free DOX, the lower cytotoxicity of DOX-loaded Fe_3_O_4_@PDA@ZIF-90 nanoparticles was observed owing to the gradual release of DOX from nanoparticles. However, after treatment with an AMF, the cells incubated with Fe_3_O_4_@PDA and Fe_3_O_4_@PDA@ZIF-90 nanoparticles exhibited the increase of fatality while no change in the control group and free DOX group, confirming the great magnetic hyperthermia capacity. The death of Hela cells obviously increased with the increase of the magnetic operating frequency. More importantly, the group of DOX-loaded Fe_3_O_4_@PDA@ZIF-90 nanoparticles treated with an AMF exhibited lower cell viability than other groups, suggesting the combined therapy results in higher therapeutic efficacy compared to chemotherapy or magnetic hyperthermia alone. Furthermore, the lowest cell viability was observed to be less than 10% when cells were treated an AMF twice. All of the results indicated that biocompatible Fe_3_O_4_@PDA@ZIF-90 nanoparticles could achieve the effective delivery of drug into tumor cells for chemotherapy, the satisfactory synergistic effect of chemotherapy and magnetic hyperthermia under AMF, and the enhancement of therapeutic efficacy by increasing the operating frequency under an AMF.

## 4. Conclusions

We have synthesized a core-shell nanoplatform assembled by Fe_3_O_4_ core as magnetic heating seed and ZIF-90 shell as drug nanocarrier (Fe_3_O_4_@PDA@ZIF-90) for synergistic magnetic hyperthermia and chemotherapy. Fe_3_O_4_@PDA@ZIF-90 nanoparticles with a mean size of 200 nm can load DOX antitumor drug with a capacity of 160 μg/mg and deliver the DOX with pH-triggered release behavior. Also, Fe_3_O_4_@PDA@ZIF-90 nanoparticles exhibited good magnetic heating ability under an AMF. More importantly, the *in vitro* results showed that biocompatible Fe_3_O_4_@PDA@ZIF-90 nanoparticles can be internalized by tumor cells, and the Fe_3_O_4_@PDA@ZIF-90/DOX nanoparticles possessed synergistic magnetic hyperthermia and chemotherapy, which had more efficient antitumor efficacy than single magnetic hyperthermia or chemotherapy. In this way, with the help of the acidic tumor microenvironment-triggered antitumor drug release and the positioning control of an AMF in the lesions, the Fe_3_O_4_@PDA@ZIF-90/DOX nanoparticles could kill more tumor cells than normal cells after EPR passive targeting, to realize synergistic treatment with negligible side effects. Therefore, Fe_3_O_4_@PDA@ZIF-90 nanoparticles would be promising for tumor therapy.
